# Failure of Elevating Calcium Induces Oxidative Stress Tolerance and Imparts Cisplatin Resistance in Ovarian Cancer Cells

**DOI:** 10.14336/AD.2016.0118

**Published:** 2016-05-27

**Authors:** Liwei Ma, Hongjun Wang, Chunyan Wang, Jing Su, Qi Xie, Lu Xu, Yang Yu, Shibing Liu, Songyan Li, Ye Xu, Zhixin Li

**Affiliations:** ^1^Medical Research Laboratory, Jilin Medical University, Jilin 132013, China; ^2^Department of Histology and Embryology, Jilin Medical University, Jilin 132013, China; ^3^Department of Pathophysiology, Basic College of Medicine, Jilin University, Changchun, 130021, China

**Keywords:** calcium, oxidative stress, cisplatin resistance, ovarian cancer

## Abstract

Cisplatin is a commonly used chemotherapeutic drug, used for the treatment of malignant ovarian cancer, but acquired resistance limits its application. There is therefore an overwhelming need to understand the mechanism of cisplatin resistance in ovarian cancer, that is, ovarian cancer cells are insensitive to cisplatin treatment. Here, we show that failure of elevating calcium and oxidative stress tolerance play key roles in cisplatin resistance in ovarian cancer cell lines. Cisplatin induces an increase in oxidative stress and alters intracellular Ca^2+^ concentration, including cytosolic and mitochondrial Ca^2+^ in cisplatin-sensitive SKOV3 cells, but not in cisplatin-resistant SKOV3/DDP cells. Cisplatin induces mitochondrial damage and triggers the mitochondrial apoptotic pathway in cisplatin-sensitive SKOV3 cells, but rarely in cisplatin-resistant SKOV3/DDP cells. Inhibition of calcium signaling attenuates cisplatin-induced oxidative stress and intracellular Ca^2+^ overload in cisplatin-sensitive SKOV3 cells. Moreover, *in vivo* xenograft models of nude mouse, cisplatin significantly reduced the growth rates of tumors originating from SKOV3 cells, but not that of SKOV3/DDP cells. Collectively, our data indicate that failure of calcium up-regulation mediates cisplatin resistance by alleviating oxidative stress in ovarian cancer cells. Our results highlight potential therapeutic strategies to improve cisplatin resistance.

Ovarian cancer is a common malignant tumor of the female reproductive organs [1-3]. Current treatment strategies for ovarian cancer include surgical resection followed by chemotherapy [4, 5]--frequently with cisplatin as the primary chemotherapeutic drug [6]. However, cisplatin resistance is a major obstacle in the effective treatment of ovarian cancer, and there is an urgent need to understand the mechanism of cisplatin resistance. Cisplatin resistance is a complex process that has been linked to an imbalance in apoptotic and prosurvival signaling [7, 8]. It was recently reported that cisplatin induces reactive oxygen species (ROS) production, and also disrupts intracellular Ca^2+^ homeostasis [9-11].

Intracellular Ca^2+^ homeostasis is crucial for cell fate, and is associated with many important cellular processes such as cell proliferation, programmed cell death, muscle excitation-contraction, gene transcription, and signal transmission [12]. Ca^2+^ transport into and out of the endoplasmic reticulum or sarcoplasmic reticulum--which stores calcium--plays a key role in buffering cytosolic Ca^2+^ concentrations [13-15]. Thus, an increase in cytosolic Ca^2+^ affects mitochondrial Ca^2+^ concentrations [16]. Once mitochondrial Ca^2+^ overload occurs, a regulatory network of mitochondrial-dependent apoptosis may be activated. It has been demonstrated that cisplatin could induce an elevation of [Ca^2+^]_i_, and regulate activation of calpain, even trigger cell apoptosis in Hela cells [17]. However, it is still not clear that whether calcium is involved in cisplatin resistance. Moreover, the imbalance in intracellular Ca^2+^ induces mitochondrial ROS production [16].

Under normal cellular conditions, ROS act as signaling molecules [11]. However, ROS such as superoxide anions, hydrogen peroxide, and hydroxyl radicals induce oxidative stress, which disrupts normal physiological homeostasis and can damage cellular components [18]. During normal aerobic metabolism, the mitochondrial respiratory chain produces ROS. However, large amounts of ROS may disrupt mitochondrial function, and activate the mitochondrial apoptotic pathway. In addition, previous research suggests that excessive levels of ROS damage various cellular macromolecules, including DNA [19-21]. Cisplatin can induce the generation of ROS, which can aggravate cisplatin-induced DNA damage, or induce mitochondrial apoptotic pathway [22]. However, it is not clear whether oxidative stress induced by altered intracellular Ca^2+^ signaling is involved in cisplatin resistance of human ovarian cancer cells.

In this study, we found that the level of oxidative stress and changes in intracellular Ca^2+^ levels (including cytosolic and mitochondrial Ca^2+^) were much less pronounced in cisplatin-resistant SKOV3/DDP cells than in cisplatin-sensitive SKOV3 cells.

We show that treatment with cisplatin induces mitochondrial damage and triggers the mitochondrial apoptotic pathway in cisplatin-sensitive SKOV3 cells, but rarely in cisplatin-resistant SKOV3/DDP cells. Blocking calcium signaling attenuates cisplatin-induced oxidative stress and intracellular Ca^2+^ imbalance in cisplatin-sensitive SKOV3 cells. *In vivo*, cisplatin significantly reduced tumor growth rates in xenograft mouse models bearing tumors originating from SKOV3 cells, but not in SKOV3/DDP cells. Therefore, failure of calcium up-regulation alleviates oxidative stress, which promotes cisplatin resistance in human ovarian cancer cells.

## MATERIALS AND METHODS

### Cell culture

The cisplatin-sensitive human ovarian cancer cell lines SKOV3 and the cisplatin-resistant clone SKOV3/DDP were obtained from the Chinese Academy of Medical Sciences and the Peking Union Medical College, respectively. Cells were cultured at 37°C with 5% CO_2_ in RPMI-1640 (Gibco, Carlsbad, CA, USA) supplemented with 10% fetal bovine serum (Invitrogen, Carlsbad, CA, USA). SKOV3/DDP cells were maintained in the same medium containing 1 µg/mL cisplatin (Sigma, St. Louis, MO, USA) to maintain the multidrug-resistant phenotype.

### Cell viability assays

We measured cell viability by the 3-(4,5-dimethylthiazol-2-yl)-2, 5-diphenyltetrazolium bromide (MTT) assay. SKOV3 cells and SKOV3/DDP cells were seeded into 96-well culture plates in 100 µL medium at a density of 1×10^4^ cells/well. Two days later, agents were added to quadruplicate wells and incubated for 24 or 48 h. For MTT assays (Beyotime, Shanghai, China), 20 µL/well MTT (5 mg/mL in phosphate-buffered saline [PBS]) was was injected into each well; 4 h later, dimethyl sulfoxide (150 µL/well; Beijing Chemical Industry, Beijing, China) was added and plates were shaken at room temperature for 10 min. Absorbance was measured at wave length of 570 nm using a Microplate Reader (BioTek Instruments, Winooski, VT, USA).

### Western blot analysis

SKOV3 and SKOV3/DDP cells were harvested after drug treatment. After washing cells with cold PBS, RIPA was added to isolate total proteins.

Cell lysates were sonicated, incubated for 15 min on ice, and clarified at 700 g for 10 min at 4°C. The supernatant was centrifuged at 14,000 g for another 30 min at 4°C; cytoplasmic proteins were present in the supernatant. The concentration of proteins was measured by the Bio-Rad protein assay (Bio-Rad Laboratories, Hercules, CA, USA). For western blot analysis, protein samples (30-50 µg for each group) were resolved by 8%, 10%, or 15% SDS-polyacrylamide gel electrophoresis and transferred onto Immobilon-P membranes (EMD Millipore, Billerica, MA, USA). Membranes were blocked in buffer (100 mM NaCl, 10 mM Tris-HCl, pH 7.6, and 0. 1% Tween 20) containing 5% non-fat dry milk for 1.5 h at room temperature and incubated with the relevant primary antibody overnight at 4°C. Membranes were then incubated with secondary antibodies at room temperature for 1.5 h.

Immunodetection was performed using enhanced chemiluminescence reagents (Thermo Scientific, Rockford, IL, USA) and images were visualized using a Syngene bioimaging system (Synoptics, Cambridge, UK). Specific proteins were quantified by densitometry using Quantity One software (Bio-Rad Laboratories), normalized to actin, and presented as the mean ± SD of three independent experiments.

### Detection of intracellular reactive oxygen species

Intracellular cisplatin-induced ROS levels were measured by staining with dichlorodihydrofluorescein diacetate (DCFH-DA), which is cell permeable and interacts with intracellular ROS to generate fluorescent dichlorofluorescin [23]. SKOV3 cells and SKOV3/DDP cells were plated at 5×10^4^ cells per well in 24-well plates, and cultured with cisplatin for 8 or 16 h. After preincubation, the medium was discarded and the attached cells were rinsed three times with PBS. SKOV3 cells and SKOV3/DDP cells were then exposed to 5 µM DCFH-DA solution for 15 min at 37°C. After treatment, cells were washed three times with PBS. Subsequently, cells were scanned to quantitate the average fluorescence intensity per cell by confocal laser microscopy.

### Calcium concentration analysis and cell death assay

Using confocal laser microscopy, Ca^2+^ concentration was determined by staining with Ca^2+^ sensitive fluorescent dyes Fluo-4/AM (Molecular Probes, Eugene, OR, USA) and Rhod-2/AM (AAT Bioquest, Sunnyvale, CA, USA). Cells were incubated with Fluo-4/AM or Rhod-2/AM for 30 min at 37°C prior to treatment under different experimental conditions. All experiments were performed in triplicate. Cell morphology was detected using an Apoptosis and Necrosis Assay Kit (Beyotime Institute of Biotechnology, Shanghai, China). Following treatment with cisplatin, both cell lines were stained with Hoechst 33342 and propidium iodide according to the manufacturer’s instructions, and then examined by confocal laser microscopy. Necrotic cells stain bright red and weak blue, appearing as purple when merged; apoptotic cells stain bright blue and weak red, indicating condensed or fragmented nuclei; and normal cells stain weak blue and weak red. The cell death rate was assessed based on a random selection of about 1,000 cells.

### Electron microscopy

Cellular morphometry was analyzed by electron microscopy. After fixing cells for 30 min with ice-cold 2.5% glutaraldehyde in 0.1 M cacodylate buffer, cells were processed for transmission electron microscopy by standard procedures. After ultra-thin sectioning, cells were examined using a transmission electron microscope at a magnification of 1,500X.

### Flow cytometry analysis

Cell death was detected using the MUSE™ Annexin-V Dead Cell kit (EMD Millipore Corporation, Hayward, CA, USA). In our experiments, SKOV3 and SKVO3/DDP cells were seeded in 6-well culture plates at a density of 2×10^5^ cells/well. After treatment under different experimental conditions, cells were trypsinized and resuspended in RPMI-1640 medium with 10% FBS at a concentration of 1×10^6^ cells/mL. Under darkroom conditions, cells were incubated with Annexin-V at room temperature for 20 min. Finally, cells were measured using the MUSE™ Cell Analyzer (EMD Millipore Corporation, Hayward, CA, USA). All experiments were performed in triplicate.

### Ovarian cancer tumor xenograft in female nude mice

Athymic BALB/c female nude mice (4 weeks old, weight 20g) were purchased from Vital River Laboratory Animal Technology Co. Ltd, Beijing, China. Female nude mice were maintained in specific pathogen-free conditions and environment under controlled conditions of 12-h light/12-h dark cycle for three days. A xenograft model of human ovarian cancer in female nude mice was established through subcutaneously injecting SKOV3 or SKOV3/DDP cells into the right of each mouse. The mice were randomly divided into 4 groups, which were the control group of SKOV3 cells, the cisplatin group of SKOV3 cells, the control group of SKOV3/DDP cells, and the cisplatin group of SKOV3/DDP cells. Treatment was started when the tumour reached about 150 mm^3^-200mm^3^. 2mg/ml cisplatin was intraperitoneally injected once two days. Tumor diameter was measured using a caliper every 2 days, and the tumor volume was calculated according to the formula: length × width × height × 0.5. After treatment with cisplatin, the tumors were excised.

### Statistical Analysis

Data are representative of three independent experiments performed in triplicate and presented as the means ± SD. Data analysis was performed using one-way analysis of variance. Tukey’s post-hoc test was used to determine statistical significance of all pairwise comparisons of interest; p<0.05 was considered statistically significant.

**Figure 1.**
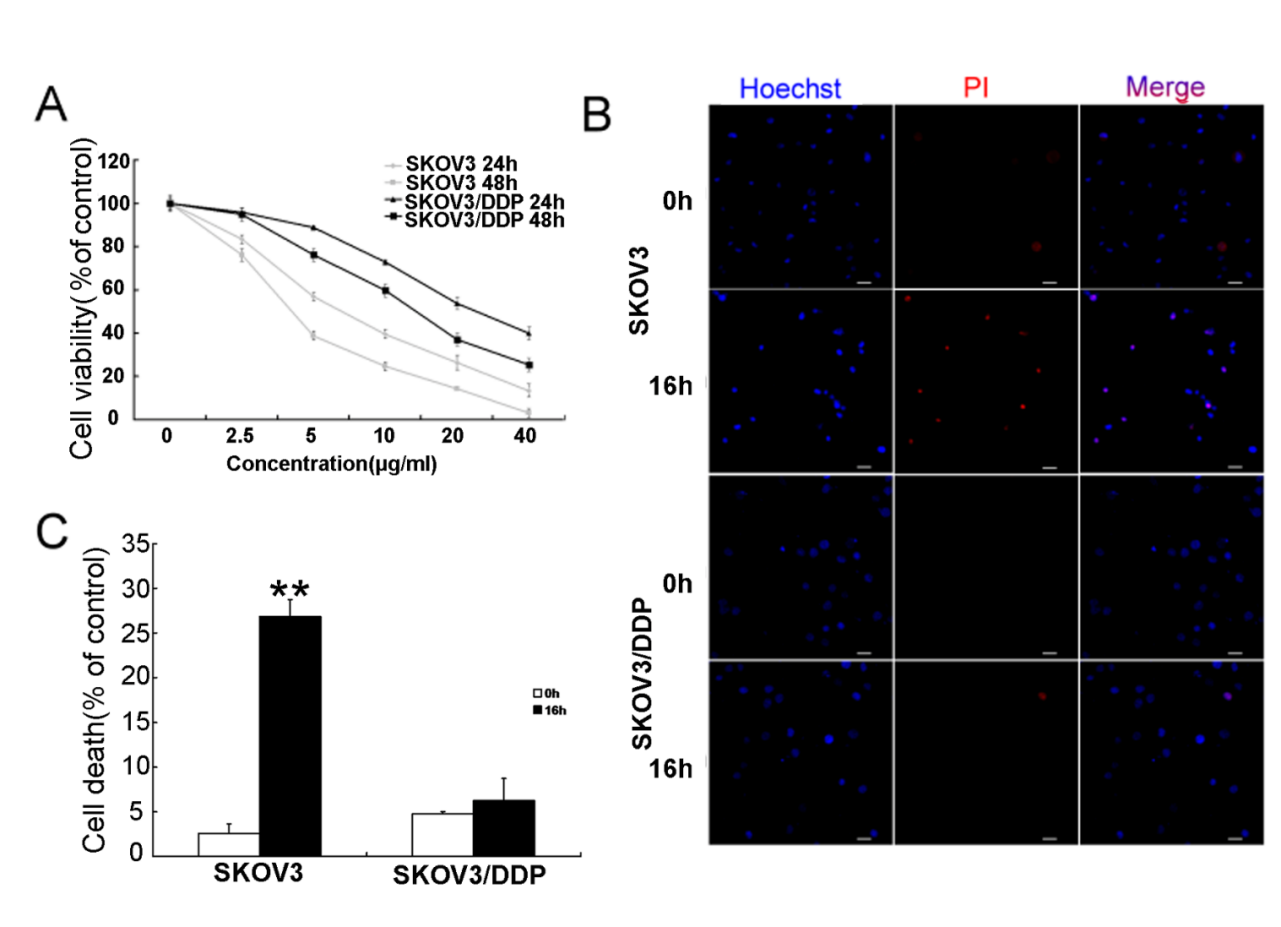
Cisplatin inhibits proliferation and induces cell death of ovarian cancer cells (A) SKOV3 and SKOV3/DDP cells were treated with varying doses of cisplatin for 24 or 48 h. Cell viability was determined by the MTT assay. Data are presented as means ± SD, n=3. (B) Cells were treated with 6 μg/ml cisplatin for 0 h and 16 h, and then stained with Hoechst 33342 and PI. Then, cells were observed by confocal microscopy (bar, 20 μm). (C) Quantitation of cell death ratio. Data are presented as means ± SD, n=3, **P<0.01 vs. control.

## RESULTS

### Cisplatin inhibits cell viability and results in cell death of ovarian cancer cells

To evaluate the effect of cisplatin on ovarian cancer cells, increasing doses of cisplatin were applied to cisplatin-sensitive SKOV3 cells and cisplatin-resistant SKOV3/DDP cells for 24 h and 48 h. According to the MTT assay, cisplatin inhibited cell viability of both cell lines, but more so in SKOV3 cells (Fig. 1A).

With reference to the MTT results and previous studies [24], both cell lines were treated with 6 µg/mL cisplatin, and stained with Hoechst 33342. As shown in Fig. 1B and C, compared with controls at 16 h, the ratio of cell death is significantly higher in cisplatin-sensitive SKOV3 cells than cisplatin-resistant SKOV3/DDP cells. These results indicate that the cytotoxicity of cisplatin is more apparent in SKOV3 cells than SKOV3/DDP cells.

**Figure 2.**
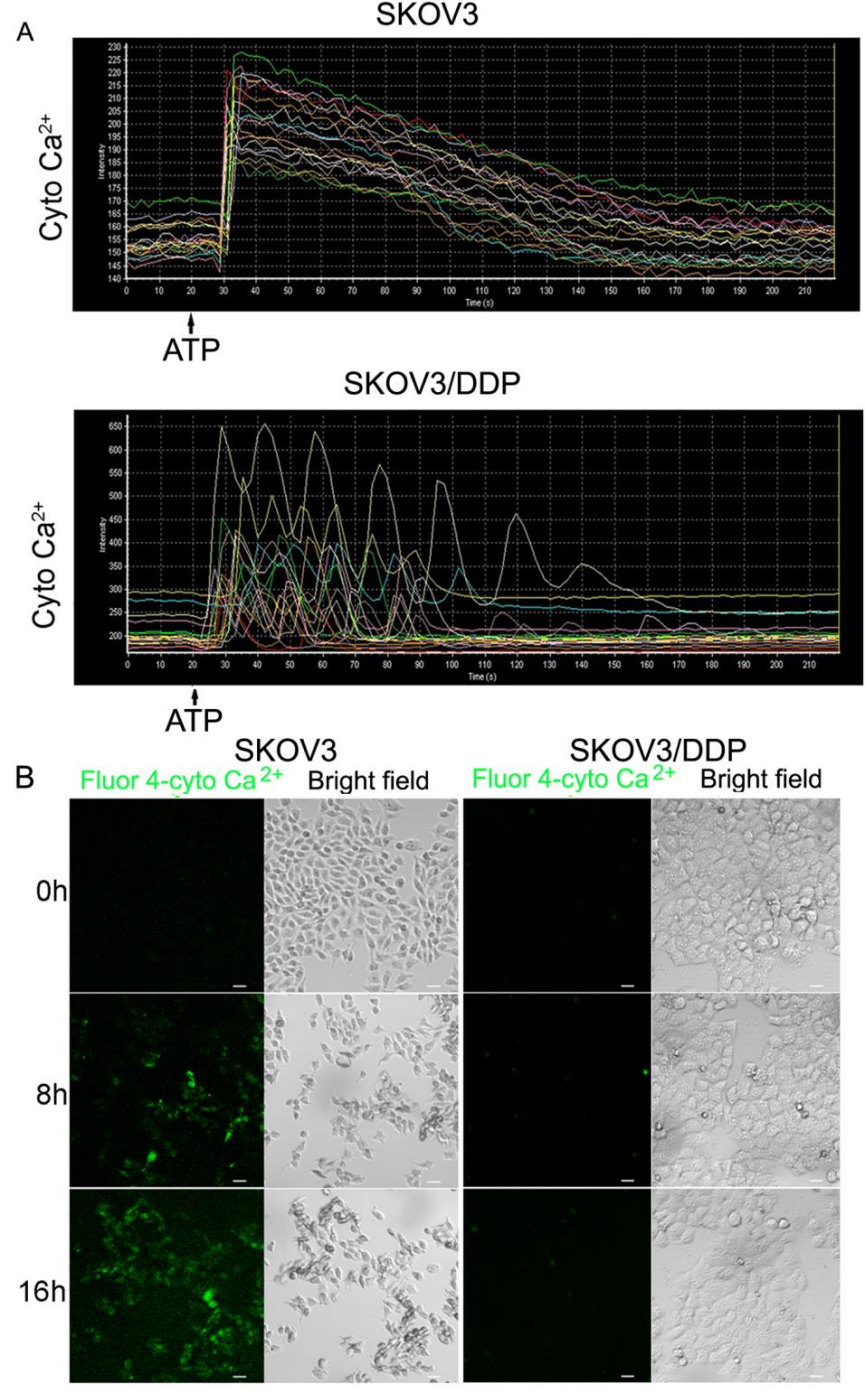
Alteration of cytosolic Ca^2+^ is significantly induced in SKOV3 cells by cisplatin or ATP, but not in SKOV3/DDP cells (A) Cells were treated with ATP and time-lapse imaging was used to detect changes in cytosolic Ca^2+^ levels. Data were obtained by confocal laser microscopy. (B) After cells treated with 6 μg/mL cisplatin for 0 h, 8 h and 16 h, cytosolic Ca^2+^ levels was detected by Fluo-4/AM (bar, 20 μm).

### Cisplatin or ATP treatment-induced an increase of cytosolic Ca^2+^ is more efficient in SKOV3 cells than SKOV3/DDP cells

Ca^2+^ homeostasis is crucial for cell survival, and influences a variety of cellular processes, such as metabolism, cell proliferation, gene transcription, programmed cell death, and signal transmission [12]. Thus, we assessed the change in cytosolic Ca^2+^ levels by Fluo-4/AM, a calcium-sensitive fluorescent probe. As shown in Fig. 2A, ATP treatment resulted in a continuous dramatic rise in cytosolic Ca^2+^ in SKOV3 cells, which presented an obvious peak. However, ATP treatment only induced a continuous weak oscillation in SKOV3/DDP cells, which showed some transient smaller fluctuations. These might be associated with cisplatin resistance of SKOV3/DDP cells.

Next, we investigated whether cisplatin influenced the level of cytosolic Ca^2+^ in both cell lines. We found that cytosolic Ca^2+^ was notably increased in SKOV3 cells, but not in SKOV3/DDP cells, both treated with cisplatin for 8 and 16 h (Fig. 2B).

Based on these results, we conclude that cytosolic Ca^2+^ overload is relatively more efficient in SKOV3 cells than SKOV3/DDP cells following treatment with ATP or cisplatin.

**Figure 3.**
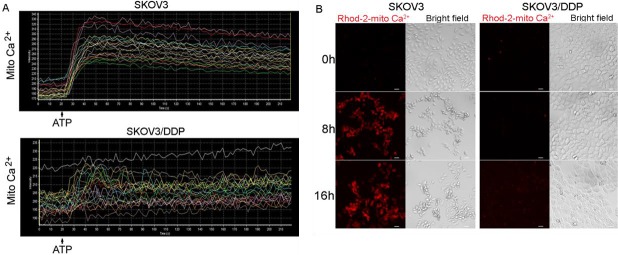
Cisplatin or ATP treatment induces mitochondrial Ca^2+^ influx in SKOV3, but not SKOV3/DDP cells (A) Both cell lines were treated with ATP and time-lapse scanning was used to detect the changes of mitochondrial Ca^2+^. Data were obtained by confocal laser microscopy. (B) After cells treated with 6 μg/ml cisplatin for 0 h, 8 h and 16 h, mitochondrial Ca^2+^ was detected by confocal microscopy (bar, 20 μm).

### Cisplatin or ATP-induced an increase of mitochondrial Ca^2+^ overload is more efficient in SKOV3 cells than SKOV3/DDP cells

Once local cytoplasmic Ca^2+^ concentrations rise, calcium is transported into mitochondria, which may result in mitochondrial Ca^2+^ overload. We investigated the change in mitochondrial Ca^2+^ by Rhod-2/AM, a calcium-sensitive fluorescent probe. After ATP treatment in SKOV3 cells, mitochondrial Ca^2+^ was increased dramatically and continually in SKOV3 cells, which resulted in an obvious peak. However, ATP-induced weakly oscillation did not form obvious peaks in SKOV3/DDP cells (Fig. 3A). These might suggest mitochondrial Ca^2+^ homeostasis plays a key role in cisplatin resistance of SKOV3/DDP cells.

Next, we investigated whether cisplatin triggers an increase in mitochondrial Ca^2+^ levels in both cell lines and found a substantial increase in SKOV3, but not in SKOV3/DDP cells, both treated with cisplatin for 8 and 16 h (Fig. 3B).

These results suggest that mitochondrial Ca^2+^ homeostasis is relatively more efficient in SKOV3 cells than SKOV3/DDP cells following treatment with ATP or cisplatin.

**Figure 4.**
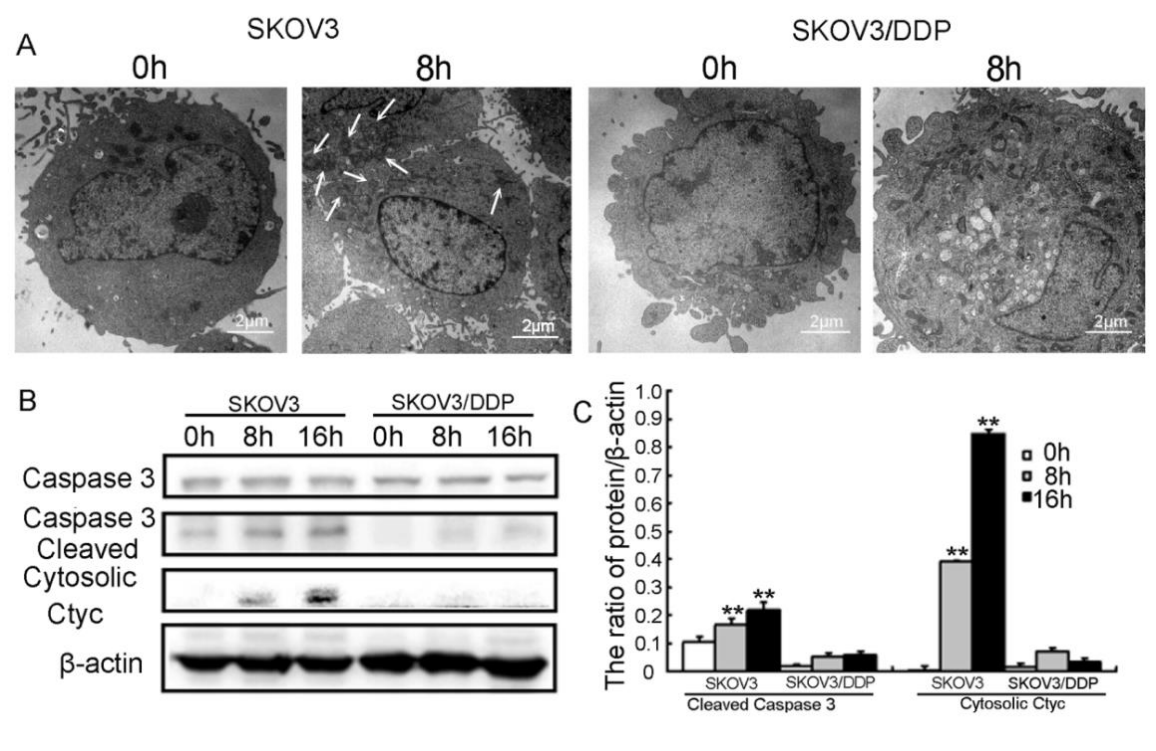
Mitochondrial Ca^2+^ overload induces cell apoptosis through mitochondrial-dependent pathway in SKOV3 cells (A) Representative transmission electron microscopy photomicrographs of both cell lines treated with 6μg/ml cisplatin for 8 h. Mitochondrial morphologies are normal in control cells (1,500x). Exposure to 6μg/ml cisplatin for 8 h resulted in mitochondrial damage (1,500x; arrows indicate mitochondrial damage). (B) Western blot analysis of cytosolic cytochrome c, caspase-3, and cleaved caspase-3 expression in cells treated with cisplatin for 0 h, 8 h, and 16 h. (C) Quantification of cytosolic cytochrome c and cleaved caspase-3 protein. Data are presented as means ± SD, n=3. **P<0.01 vs. control.

### Mitochondrial Ca^2+^ overload induces cell apoptosis through the mitochondrial-dependent pathway in SKOV3 cells

The mitochondrion is the key energy-producing cytoplasmic organelle and an important regulator of metabolic homeostasis [25]. Mitochondrial Ca^2+^ overload destroys normal mitochondrion morphology and results in mitochondrial-dependent apoptosis [26]. Therefore, we determined ultrastructural changes in the mitochondria of both cell lines via electron microscopy. Following treatment with cisplatin, the mitochondria of SKOV3 cells had various morphologies, such as swelling or vacuolar and even fractured, compared with controls, which was rarely seen in SKOV3/DDP cells (Fig. 4A). Mitochondria play a crucial role in apoptosis [27]. Thus the damage of mitochondria might induce apoptosis, and caspase 3 regulates mitochondrial events in the execution phase of apoptosis and in the initial phase of apoptosis such as cytochrome c release [27, 28]. Next, we assessed the expression of apoptosis-associated proteins such as caspase-3 and cytosolic cytochrome c through western blotting. Following treatment with cisplatin, cleaved caspase-3 and cytosolic cytochrome c were detected at 8 h and 16 h in SKOV3 cells, but not in SKOV3/DDP cells (Fig. 4B and C).

Based on these results, we conclude that cisplatin leads to mitochondrial-dependent apoptosis by inducing mitochondrial Ca^2+^ overload in SKOV3 cells.

### Inhibition of cisplatin-induced cytosolic Ca^2+^ influx and mitochondrial Ca^2+^ overload reduces intracellular ROS production in SKOV3 cells

To evaluate whether the homeostasis of cytosolic Ca^2+^ and mitochondrial Ca^2+^ is involved in regulating intracellular ROS production in ovarian cancer cells, we treated ovarian cancer cell lines with a combination of cisplatin, the calcium chelating agent bis-(o-aminophenoxy)ethane-N,N,N′,N′-tetra-acetic acid acetoxymethyl ester (BAPTA/AM), and inositol triphosphate receptor (IP3R) inhibitor 2-Aminoethyl diphenylborinate (2-APB).

**Figure 5.**
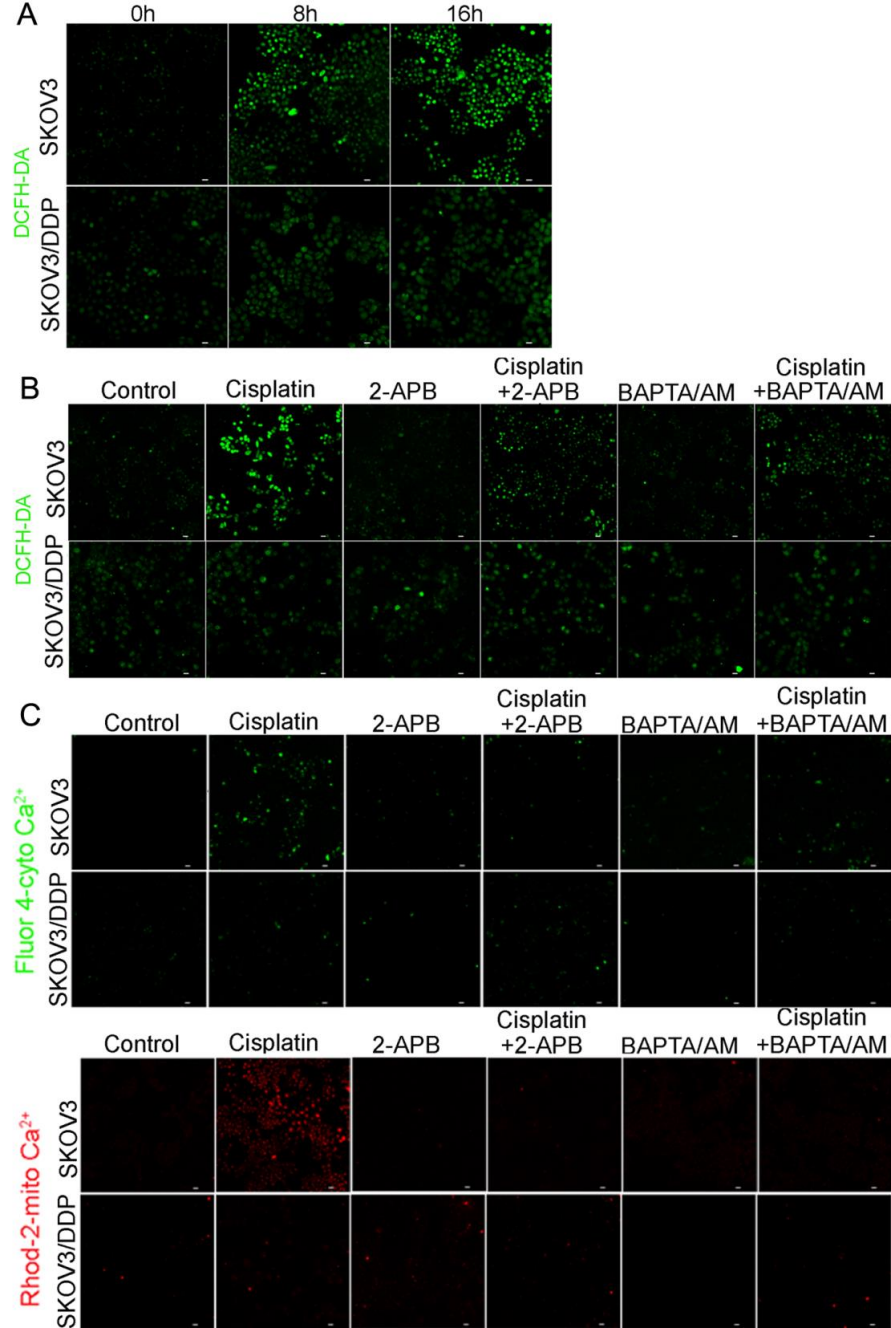
Inhibition of cisplatin-induced cytosolic Ca^2+^ influx and mitochondrial Ca^2+^ overload reduces intracellular ROS production in SKOV3 cells (A) Cells were treated with 6 μg/ml cisplatin for 0 h, 8 h and 16 h, and stained with DCFH-DA (bar, 20 μm). (B) After cells treated with 6 μg/ml cisplatin with or without 2-APB (50 μM) and BAPTA/AM (30 μM) for 16 h, cells were stained with DCFH-DA (bar, 20 μm). (C) After the same treatment with (A), cytosolic Ca^2+^ levels were detected by Fluo-4/AM and mitochondrial Ca^2+^ was detected by Rhod-2/AM (bar, 20 μm).

We found that ROS levels increased significantly in SKOV3 cells 8 h and 16 h after cisplatin treatment (using DCFH-DA staining), but only slightly in SKOV3/DDP cells (Fig. 5A). Meanwhile, following treatment with a combination of cisplatin and BAPTA/AM or 2-APB, intracellular ROS levels did not increase in SKOV3 cells relative to the no treatment group (Fig. 5B); this treatment did not however affect intracellular ROS production in SKOV3/DDP cells (Fig. 5B). As an intracellular Ca^2+^ chelator, the addition of BAPTA/AM decreased cisplatin-induced cytosolic Ca^2+^ influx. Meanwhile, the addition of 2-APB, an inhibitor for inositol 1,4,5-trisphosphate (IP3) receptors, reduced mitochondrial Ca^2+^ overload in SKOV3 cells, but not in SKOV3/DDP cells (Fig. 5C). These showed BAPTA/AM or 2-APB prevented imbalance of cisplatin-induced Ca^2+^ homeostasis, and inhibited cisplatin-induced generation of ROS.

These results suggest that alteration of cisplatin-induced cytosolic and mitochondrial Ca^2+^ homeostasis affects intracellular ROS production in SKOV3 cells, but cisplatin does not affect Ca^2+^ homeostasis and ROS level in SKOV3/DDP cells.

**Figure 6.**
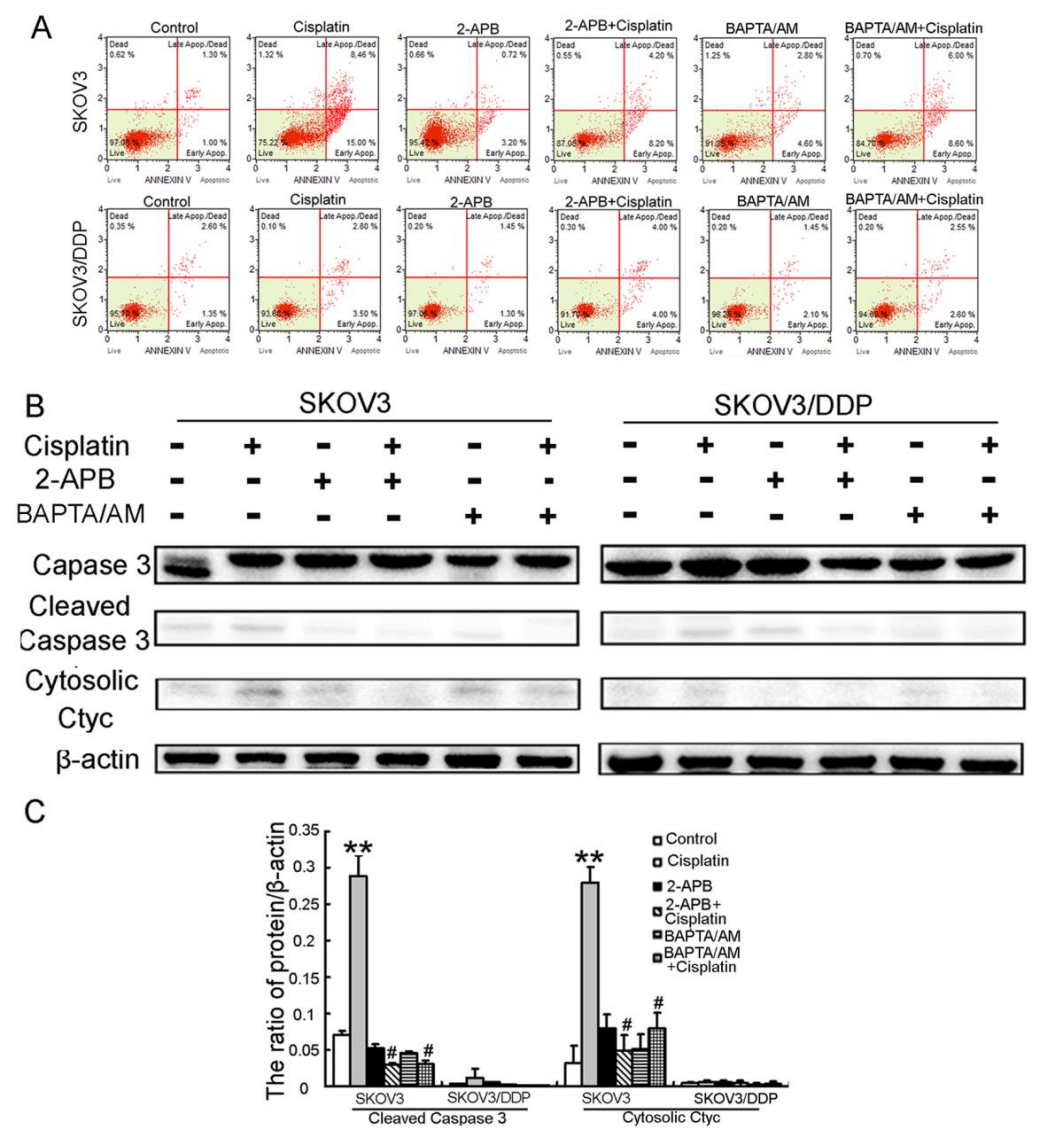
Inhibition of cisplatin-induced cytosolic Ca^2+^ influx and mitochondrial Ca^2+^ overload protect SKOV3 cells from cisplatin-induced apoptosis (A) After cells treated with 6 μg/ml cisplatin with or without 2-APB (50 μM) and BAPTA/AM (30μM) for 16 h, cells were then stained with Annexin-V. Data are presented as the mean ± SD, n = 3. (B) After the same treatment with (A), the expression of caspase-3, cleaved caspase-3, and cytosolic cytochrome c in both cell lines is detected by western blotting. (C) Quantitation of cleaved caspase-3, and cytosolic cytochrome c protein levels. Data are presented as the mean ± SD, n = 3. **P < 0.01 vs. control, ^#^*P* < 0.05 vs. cisplatin.

### Inhibition of cisplatin-induced cytosolic Ca^2+^ influx and mitochondrial Ca^2+^ overload protect SKOV3 cells from cisplatin-induced apoptosis.

To better determine the role of calcium in cisplatin-induced apoptotic cell death, ovarian cancer cell lines were treated with a combination of cisplatin and BAPTA/AM or 2-APB. Cisplatin significantly increased the rate of apoptosis in SKOV3 cells compared with the control, but not in SKOV3/DDP cells (Fig. 6A). Meanwhile, cisplatin combined with BAPTA/AM or 2-APB significantly decreased the rate of apoptosis in SKOV3 cells compared with cisplatin treatment alone, but not in SKOV3/DDP cells (Fig. 6A). Next, the expression of apoptosis-associated proteins was assessed in both cell lines via western blotting. When SKOV3 cells were treated with a combination of cisplatin and BAPTA/AM or 2-APB, the expression of cleaved caspase-3 and cytosolic cytochrome c was lower than in cells treated with cisplatin alone (Fig. 6B and C). Using the same treatment, there was no change in the expression of apoptosis-associated proteins of SKOV3/DDP cells (Fig. 6B and C). These indicate that inhibition of cisplatin-induced cytosolic Ca^2+^ influx and mitochondrial Ca^2+^ overload prevents cisplatin from inducing apoptosis in SKOV3 cells.

These suggest alteration of Ca^2+^ homeostasis plays a crucial role in cisplatin-induced apoptosis.

**Figure 7.**
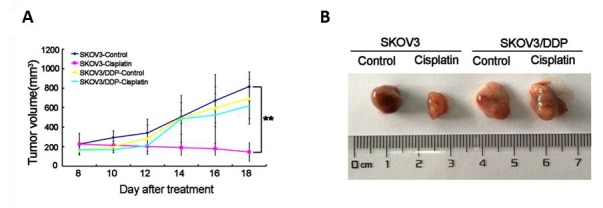
Cisplatin displays anti-tumor activity in xenograft mouse models bearing tumors originating from SKOV3 cells, but not SKOV3/DDP cells (A) The average volume of tumor originating from SKOV3 cells were obviously diminished by cisplatin, but not in SKOV3/DDP cells. (B) BALB/c femal nude mice subcutaneous transplant tumor model was established using SKOV3 and SKOV3/DDP cells. After treatment with cisplatin, tumors in mice were excised and photographed for each group. Data are presented as the mean ± SD, n = 3. **P < 0.01 vs. control.

### Cisplatin displays anti-tumor activity in xenograft mouse models bearing tumors originating from SKOV3 cells, but not SKOV3/DDP cells.

To further examine anti-ovarian cancer effect of cisplatin *in vivo*, a human ovarian cancer-nude mouse model was established by subcutaneous xenograft SKOV3 and SKOV3/DDP cell lines. 2mg/kg cisplatin decreased the tumor burden of SKOV3 cells group, but not SKOV3/DDP cells group (control vs cisplatin, **p<0.01) (Fig. 7A). When the mice were sacrificed, the tumor volume in the cisplatin group of SKOV3 cells became obviously smaller than that in the control group, but not significantly change in SKOV3/DDP cells groups (Fig. 7B). This indicated that the inhibitory effect of cisplatin was obvious in xenograft mouse models bearing tumors originating from SKOV3 cells, but not SKOV3/DDP cells.

Thus, these results suggest that cisplatin inhibits the growth of the tumors originating from SKOV3 cells *in vivo*, but not SKOV3/DDP cells.

## DISCUSSION

Cisplatin is widely used for the treatment of solid tumors, such as cholangiocarcinoma [29, 30], cervical cancer [30], and ovarian cancer [24]. Cisplatin induces cell apoptosis through DNA damage and inhibition of DNA synthesis [31]. Besides binding to DNA, cisplatin is associated with the reorganization of the actin cytoskeleton, motile behavior and biochemical signatures [32]. Moreover, membrane permeability of the cell is also involved in cisplatin resistance, such as remarkably toxicity of smaller cisplatin-loaded liposomes in cancer cells [33]. At present, the therapeutic effectiveness of cisplatin is limited by its resistance in cancers, including ovarian cancer [4, 5]. Therefore, there is an urgent need to increase our understanding of cisplatin resistance in cancer. Previous mechanistic studies have focused on the modulation of intracellular Ca^2+^ homeostasis and oxidative stress in cancer cells treated with cisplatin [11, 34]. However, it is not clear whether loss of calcium overload and oxidative stress tolerance is involved in cisplatin resistance.

Our results demonstrate that SKOV3/DDP cells are more resistant to cisplatin than SKOV3 cells, as judged by cell proliferation and cell survival assays in vitro and tumor growth rates *in vivo* (Fig 1 and 7). Reports show that in fact only about 1% of intracellular cisplatin affects nuclear DNA; in addition, cisplatin also induces apoptosis in enucleated cells [35, 36]. In non-nuclear cells, ER might be a targeted organelle of cisplatin [35]. The ER not only participates in protein biosynthesis, but also maintains intracellular Ca^2+^ homeostasis [37-39]. Thus, cisplatin triggers apoptosis through altering Ca2+ homeostasis and calpain activation [35]. In our study, we show that cisplatin triggers a sharp increase in cytosolic and mitochondrial Ca^2+^ as well as mitochondrial-dependent apoptosis in cisplatin-sensitive SKOV3 cells. In cisplatin-resistant SKOV3/DDP cells, however, cisplatin does not affect intracellular Ca^2+^ homeostasis. At present, there are only a few reports that have illustrated that intracellular Ca^2+^ homeostasis may be involved in cisplatin resistance [40, 41]. The change in mitochondrial Ca^2+^ concentration greatly depends on the rise in local cytoplasmic Ca^2+^ concentrations. More importantly, a sharp increase in cytosolic Ca^2+^ not only leads to a collapse of the proton gradient and bioenergetic catastrophe, but also induces Ca^2+^ to cross mitochondrial membranes into mitochondria [12, 15, 26]. Thus, mitochondrial Ca^2+^ overload results in mitochondrial damage and induces cell apoptosis by the mitochondrial-dependent pathway [26, 42]. Our study reveals that cisplatin induces the expression of apoptotic proteins of the mitochondrial-dependent pathway in cisplatin-sensitive SKOV3 cells, but not in cisplatin-resistant SKOV3/DDP cells. Therefore, failure of calcium up-regulation may well be associated with cisplatin resistance in ovarian cancer cells.

Recent studies have reported that cisplatin leads to mitochondrial damage, including reducing the activity of respiratory complexes (I-IV) and changing mitochondrial membrane potential [43, 44], blocking mitochondrial energy production [45], altering the mitochondrial ultrastructure, lowering antioxidant capacity [46], and up-regulating the level of oxidative stress by increasing ROS production [34, 47, 48]. Notably, generation of excessive ROS leads to oxidative damage such as accentuating cisplatin-induced DNA damage or triggering apoptosis of mitochondrial-dependent pathway [22, 49]. Our results show that cisplatin induces a significant increase in ROS levels in cisplatin-sensitive SKOV3 cells, but not in cisplatin-resistant SKOV3/DDP cells. Coincidently, enhanced antioxidant capacity limits the amount of reactive cisplatin and is involved in the context of cisplatin resistance [22]. Therefore, tolerance to oxidative stress is apparently involved in cisplatin resistance in ovarian cancer cells.

An imbalance in Ca^2+^ homeostasis leads to a series of pathological conditions, such as cardiovascular disorders, neurodegenerative diseases, and cancer [50]. Moreover, Ca^2+^ signaling is associated with many tumorigenic pathways, and deregulation of Ca^2+^ homeostasis decreases cellular proliferation and leads to cell apoptosis [51-53]. Importantly, disruption of cytosolic Ca^2+^ homeostasis triggers mitochondrial ROS production [16]. The generation of excessive ROS even induces apoptosis in HepG2 cells [54]. Our results show that blocking calcium signaling attenuates cisplatin-induced intracellular Ca^2+^ and ROS production in SKOV3 cells, and that the maintenance of intracellular Ca^2+^ homeostasis protects SKOV3 cells from cisplatin-induced apoptosis. In conclusion, our study demonstrates that failure of elevating calcium mediates cisplatin resistance by alleviating oxidative stress in ovarian cancer cells.
